# A Pushed Descending Aorta due to Hiatal Hernia

**DOI:** 10.1055/s-0039-1679910

**Published:** 2019-03-08

**Authors:** Cetin Gecmen, Muzaffer Kahyaoglu, Arzu Kalayci, Ender Ozgun Cakmak, Ozkan Candan, Ahmet Guner, Ibrahim Akin Izgi, Cevat Kirma

**Affiliations:** 1 Department of Cardiology, Kartal Kosuyolu Heart and Research Hospital, Istanbul, Turkey

**Keywords:** descending aorta, aortic displacement, hiatal hernia

## Abstract

A 91-year-old female patient was admitted to hospital for evaluation of transcatheter aortic valve implantation. A chest radiography showed a hiatal hernia, and the stomach and duodenum appeared in the thoracic cavity. The descending aorta was pushed by the stomach and duodenum.

A 91-year-old female patient was admitted to hospital for evaluation of transcatheter aortic valve implantation. A chest radiography showed a hiatal hernia, and the stomach and duodenum appeared in the thoracic cavity ( Fig. 1A ). The patient had severe aortic stenosis, and a contrast-enhanced thoracoabdominal computed tomography (CT) was performed to evaluate vascular structures. A contrast-enhanced CT scan of the chest in the coronal plane revealed normal diameter and non-tortuous ascending aorta ( Fig. 1B ). The descending aorta was pushed by the stomach and duodenum ( Fig. 1C ). A contrast-enhanced CT scan of the chest in the sagittal plane showed the pushed descending aorta ( Fig. 1D ). A contrast-enhanced CT scan of the chest in axial plane showed the descending aorta on the left side of the vertebra ( Fig. 1E ). The abdominal aorta travels on the left side of the vertebra ( Fig. 1F ). A successful intervention was done, and the patient was discharged from the hospital.

**Fig. 1:**
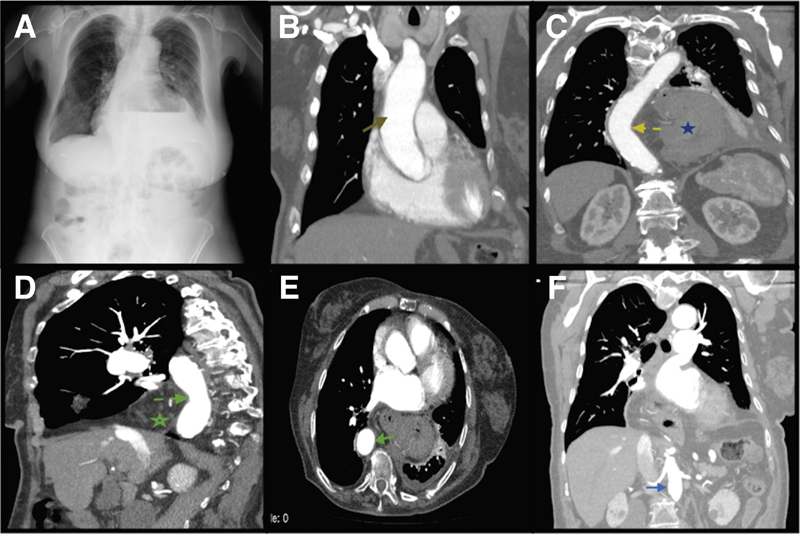
( **A** ) A chest radiography showing hiatal hernia and stomach in the thoracic cavity. ( **B** ) A contrast-enhanced computed tomography (CT) scan of the chest in coronal plane showing non-tortuous ascending aorta. ( **C** ) A contrast-enhanced CT scan of the chest in coronal plane showing the pushed descending aorta. ( **D** ) A contrast-enhanced CT scan of the chest in sagittal plane showing the pushed descending aorta. ( **E** ) A contrast-enhanced CT scan of the chest in axial plane showing the descending aorta on the left side of the vertebra. ( **F** ) Abdominal aorta travels on the left side of the vertebra.

